# Societal costs before and up to 1 year after the first fracture liaison service visit in patients requiring anti-osteoporosis treatments

**DOI:** 10.1007/s11657-024-01390-7

**Published:** 2024-05-13

**Authors:** Lieke Maas, Annelies Boonen, Caroline E. Wyers, Sandrine Bours, Joop P. van den Bergh, Silvia M. Evers, Sander M. J. van Kuijk, Mickaël Hiligsmann

**Affiliations:** 1 Department of Health Services Research, Care and Public Health Research Institute (CAPHRI), Maastricht University, P.O Box 616, 6200 MD Maastricht, The Netherlands; 2 Department of Internal Medicine, Division of Rheumatology, Maastricht University Medical Center, Maastricht, The Netherlands; 3 Department of Internal Medicine, VieCuri Medical Center, Venlo, The Netherlands; 4 Department of Internal Medicine, NUTRIM, Maastricht University Medical Center, Maastricht, The Netherlands; 5 Department of Internal Medicine, Division of Rheumatology, Maastricht University Medical Center, Maastricht, The Netherlands; 6 Department of Clinical Epidemiology and Medical Technology Assessment, Maastricht University Medical Center, Maastricht, The Netherlands

**Keywords:** Societal costs, Fracture liaison service, Osteoporosis, Fracture, Anti-osteoporosis treatment, Longitudinal study

## Abstract

**Summary:**

This study aimed to estimate societal and healthcare costs incurred before and 1 year after the first fracture liaison services (FLS) visit and to explore differences in fracture type. All costs after 1 year significantly decreased compared to costs preceding the first visit. Fracture type did not significantly affect costs.

**Introduction:**

Limited literature is available on resource utilization and costs of patients visiting fracture liaison services (FLS). This study aimed to estimate the societal and healthcare costs incurred by patients with a recent fracture requiring anti-osteoporosis medication before and 1 year after the first FLS visit and to explore differences according to fracture type.

**Methods:**

Resource utilization was collected through a self-reported questionnaire with a 4-month recall on health resource utilization and productivity losses immediately following the first FLS visit, and 4 and 12 months later. Unit costs derived from the national Dutch guideline for economic evaluations were used to compute societal and healthcare costs. Linear mixed-effect models, adjusted for confounders, were used to analyze societal and healthcare costs over time as well as the effect of fracture type on societal and healthcare costs.

**Results:**

A total of 126 patients from two Dutch FLS centers were included, of whom 72 sustained a major fracture (hip, vertebral, humerus, or radius). Societal costs in the 4 months prior to the first visit (€2911) were significantly higher compared to societal costs 4 months (€711, *p*-value = 0.009) and 12 months later (€581, *p*-value = 0.001). Fracture type did not have a significant effect on total societal or healthcare costs. All costs 12 months after the initial visit were numerically lower for major fractures compared to others.

**Conclusion:**

Societal and healthcare costs in the year following the first FLS visit significantly decreased compared to those costs preceding the first visit.

**Supplementary Information:**

The online version contains supplementary material available at 10.1007/s11657-024-01390-7.

## Introduction

Fracture liaison services (FLS) have been established worldwide to improve post-fracture care and osteoporosis treatment and even though the number of FLS is increasing; implementation in healthcare is variable and could be improved [1–4]. Despite increasing evidence on the effectiveness and cost-effectiveness of FLS including in the Netherlands [5–8], there is currently limited information available on the (societal) costs before and after an FLS visit. While a few prospective studies have estimated fracture costs, to our knowledge, the societal costs before and after the first FLS visit have not yet been investigated [5–7].

The Improvement of osteoporosis Care Organized by Nurses (ICON) study offers an opportunity to address the above-mentioned gaps in knowledge by collecting real-world longitudinal data on patients visiting the FLS after a recent fracture and eligible for anti-osteoporosis medication (AOM) [9]. The ICON study (trial registration number 2018–0575) was developed to test the effect of a multi-component adherence intervention (MCAI) combining a patient decision aid [10] with motivational interviewing on medication persistence compared to usual care in patients attending an FLS after a recent fracture and collected societal costs before and up to 1 year after the first FLS visit. The current study aimed to provide an overview of the societal and healthcare costs before and after the first FLS visit of patients with a recent fracture and requiring AOM receiving usual care at FLS. More specifically, costs over the past 4 months were estimated at the first FLS visit and were compared to estimates 4 and 12 months later. A second objective was to estimate the effect of fracture type on societal and healthcare costs over time.

## Methods

### Study population

This partial economic evaluation, specifically a bottom-up cost analysis, included patients aged 50 or older who visited the FLS due to a recent fracture (≤ 26 weeks) between September 2018 and July 2020, diagnosed with osteoporosis (defined as a *T*-score ≤  − 2.5) and/or a moderate/severe vertebral fracture, and had not used AOM within 12 months before inclusion. All included patients had a treatment indication of AOM based on the Dutch guideline for osteoporosis and fracture prevention [11]. Patients were excluded if they (i) had contra-indications for oral AOM; (ii) had severe comorbidities (e.g., current malignancies); (iii) did not fully understand the study; (iv) were not able to fill in the questionnaires due to language barriers; (v) or were treated with intravenous infusions (i.e., zoledronic acid). Patients who met the inclusion criteria and were interested in participating were referred to the researcher or doctor’s assistant to obtain verbal and written informed consent.

### FLS setting

For this study, patients from the usual care group of the ICON study were included. All patients attended one of two FLS centers for fracture risk evaluation [9] (Maastricht University Medical Center (MUMC +) and VieCuri Medical Center Venlo, the Netherlands). The ICON study was approved by the Medical Ethics Committee of MUMC + /Maastricht University (UM), the Netherlands, registration number 2018–0575, and the trial was registered in the Netherlands Trial Registry (Trial NL7236 (NTR7435) Version 1.0; 26–11-2020).

FLS care comprises a multidisciplinary approach, including specialists of various medical fields such as orthopedic surgeons, endocrinologists, rheumatologists, geriatricians, primary-care physicians, and nurses. FLS care consisted of an initial visit that included assessment of risk for osteoporosis and for subsequent fractures, measurement of bone mineral density by Dual-Energy X-ray Absorptiometry (DXA) and vertebral fracture assessment, and laboratory assessment. The outcomes of the assessments were discussed with the patient. Next, the patient was educated on the diagnosis and risk of subsequent fracture and was advised on interventions with regards to lifestyle and AOM to improve bone health. A follow-up visit was scheduled 3 to 4 months after the initial FLS visit and included evaluation of medication adherence and possible side effects.

### Identification and measurement

Resource utilization was collected in the past 4 months via an adjusted self-reported questionnaire, which was developed using a technique for resource consumption measurement based on patient recall [12, 13] and included healthcare consumption within the healthcare system (visits to the general practitioner, medical specialists, paramedics including those providing mental health care, or hospital administration), utilization for the patient and their relatives (receiving home care or care from a caregiver), and resources from other sectors outside the healthcare sector (productivity losses of paid and/or unpaid work). Patients completed the questionnaire after the first FLS visit, and 4 and 12 months later.

Information on the initial fracture type, whether the patient underwent surgery for their initial fracture, and the duration between the initial fracture and the first FLS visit with the osteoporosis nurse were gathered using electronic patient files. Additional data such as date of birth, highest achieved education level (categories from lowest to highest: elementary school, lower vocational, pre-vocational secondary, general secondary, secondary vocational, higher vocational education, university), and gender were collected via the questionnaire at the first FLS visit. Fractures were classified as major fractures (hip, clinical vertebral, humerus, or radius fracture) or other fractures, based on the Major Osteoporotic Fractures classification of the International Osteoporosis Foundation [14].

### Valuation

Healthcare and productivity losses costs were based on 2022 reference values [15] and adjusted to 2023 with data from the Central Bureau of Statistics (CBS) [16]. Cost valuation was performed using the most recent version of the Dutch guideline for healthcare and productivity losses costs [15]. Given that all patients were registered in the diagnosis treatment combination (DBC) system, the average DBC price for treatment at FLS (€515 in 2023) and the average DBC price for a surgical healthcare consumption (€610 in 2023) was used. The FLS DBC entailed all diagnostic workup such as DXA scans, two visits with an osteoporosis nurse/medical specialist, and one follow-up visit with the general practitioner. The surgical DBC included diagnostics and two visits with a medical specialist. As fracture types in our study might not be generalizable due to the small sample size and lack of data on whether patients received prosthetics or endured complications from the surgery, surgery costs were not included in the study. To prevent overlap between self-reported healthcare consumption and DBC content, the two FLS visits and if applicable two follow-up visits with a surgeon from the self-reported questionnaires were not included in the cost analysis [16]. The friction cost method was applied to calculate loss of paid and unpaid work [15]. Discounting (i.e., converting future costs to relative value) was not performed as the follow-up period did not exceed 1 year.

### Data analysis

R version 4.3.1 was used for data analyses. Patient characteristics at baseline and resource utilization at each assessment point were described using descriptive statistics. To account for the uneven time intervals in the statistical models, the average monthly costs for each time point (4 months before the first FLS visit, and 4 and 12 months after) were used.

Societal costs (sum of healthcare and productivity cost) and healthcare costs separately were tested statistically. First, linear mixed-effect regressions (LMM) were used to analyze solely societal or healthcare costs over time. Second, LMM was used to analyze the overall trends and investigates the general impact of fracture type (or aggregate effect) on societal and healthcare costs, capturing the aggregate effect of fractures without focusing on specific subgroups. Third, the understanding of specific fracture subgroups over time was analyzed using a LMM. Where applicable, the best model fit was determined using the Akaike Information Criterion (AIC) [17]. LMM are presented as coefficients with its 95% confidence intervals (CI) and *p*-values. The *β*-coefficient illustrates the mean difference in costs between the first measurement of the 4 months before the first FLS visit and the tested time point.

LMM were adjusted for confounding factors, that is, (i) time between initial fracture and first FLS visit; (ii) type of fracture (major versus other); (iii) whether surgery was performed for the initial fracture; (iv) FLS center (MUMC + or VieCuri); and (v) gender. Multiple imputation according to the method described by Van Buuren et al. [18] were used to account for missing data. Thirty imputed datasets were created and predictive mean matching was used for continuous, polytomous regression for categorical variables, and logistic regression for dichotomous variables. Sensitivity analyses were performed to analyze the impact of additional potential confounding factors by including additional variables in the statistical models, namely, individual fracture types and age.

## Results

### Patient population

A total of 126 patients (94 from MUMC + and 32 from VieCuri) were included in the analyses. The mean age at initial FLS visit was 71 ± 10 years (Table 1) and 100 patients were female (79%). The average time between initial fracture and first FLS visit was 3.8 ± 2.6 months. In total, 72 patients had a major fracture (hip: 15, vertebral: 26, humerus: 13, radius: 18) and 54 patients had other fractures (43%) (Supplementary Table 1 for patient characteristics per FLS center). The data included 1.5 to 19.7% missing data (Supplementary Table 2). Of the 72 patients with a major fracture, 19 (26%) underwent surgery of which 7 more than 4 months to the first FLS visit. Additionally, 13/52 patients (24%) with other fractures (non-major) underwent surgery of which 5 more than 4 months before the first FLS visit. DBC costs of the surgery were included in our cost estimates independent of the time of surgery.

**Table 1:** Patients’ demographic and clinical characteristics

Patient characteristics	Mean ± SD/frequency (%)
Age (mean ± SD)	71 ± 10
Female (freq)	100 (79%)
Education — college level (higher vocational/university)	34 (27%)
FLS center: (%)	
MUMC +	94 (75%)
VieCuri	32 (25%)
Major fracture (freq)	72 (57%)
Surgery (freq)	32 (25%)
Time from fracture to first FLS (mean months ± SD)	3.8 ± 2.6
% with surgery > 4 months before first FLS	12 (10%)
Recurrent fracture during trial (freq)	17 (14%)

*SD*, standard deviation; %, percentage; *freq*, frequency; *FLS*, fracture liaison service; *MUMC+*; Maastricht University Medical Centre+

The 4-month societal costs per patient entailed on average €2911 before the first FLS visit, and decreased to €711 4 months later and to €581 12 months after the first FLS visit. Societal costs decreased significantly across all time points (*p*-value < 0.01) (Supplementary Table 3). The 4-month healthcare costs per patient entailed on average €2104 before the first FLS visit and decreased to €485 4 months later and €434 12 months after the first FLS visit (Table 2). Linear mixed models demonstrated similar significant changes in healthcare costs over time (*p*-value < 0.04) (Supplementary Table 3).

**Table 2:**
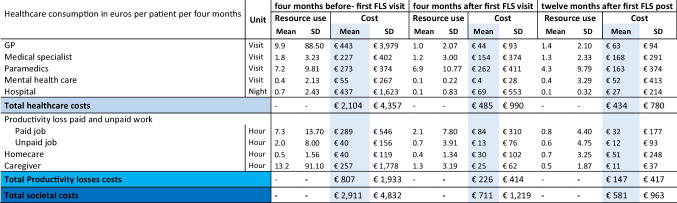
Total societal costs (dark blue) of patients (*n* = 126) before and after the first fracture liaison service visit, divided into total healthcare (light blue) and total productivity losses (bright blue)

*FLS*, Fracture Liaison Service; *SD*, standard deviation; *GP*, general practitioner or nurse assistant; *PL*, productivity loss; *OP*, osteoporosis

The stratification based on fracture type revealed that patients who experienced a major fracture had more societal and healthcare costs 4 months prior to the first FLS visit (Table 3). However, 4 and 12 months after the first FLS visit less societal and healthcare costs were seen compared to patients who experienced other fractures (non-major). Fracture type did not have a significant aggregate effect on total societal costs (*p*-value = 0.659), nor was there evidence of significant difference in fracture types over time (*p*-value > 0.4). Similar patterns and no statistical difference were observed for healthcare costs regarding fracture type (Supplementary Table 4). Sensitivity analyses did not show different results (Supplementary Table 5 and 6).

**Table 3:**
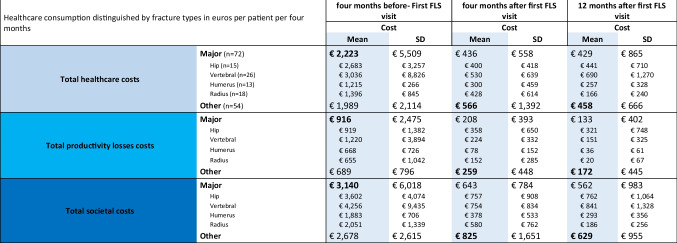
Total costs of our sample, categorized in healthcare (light blue), productivity (bright blue), and societal costs (dark blue) by major and other fracture type

One patient of the major fracture group had a pelvis fracture. *FLS*, Fracture Liaison Service; *SD*, standard deviation; *PL*, productivity loss

## Discussion

This study compared the societal costs before and after the first FLS visit in 126 patients with treatment indication who were eligible for AOM. The societal costs 4 months before the first FLS visit were estimated at €2911, and significantly decreased to €711 4 months later and to €581 one year later.

To our knowledge, this study is the first to estimate the costs before and after the first FLS visit. Previous studies have estimated fracture costs. For example, Zeelenberg et al. recently estimated the cost of hip fractures over a 5-year period in the Netherlands at €23,351 [19]. In our study, patients were asked to report their costs in the 4 months before the first FLS visit. The mean duration between the fracture and the first FLS visit was estimated at 3.6 months (SD: 2.6); therefore, our estimation did not include possible healthcare consumption and productivity losses costs other than surgery that were made in the period between 4 months before the first FLS visit and their surgery for several patients (12/126).

As expected, our study demonstrates significant decrease in both societal and healthcare costs over time. As expected, the first months after initial fracture healthcare consumption is per definition higher due to possible surgeries and additional contacts with medical specialists and paramedics. Additionally, in those few months the need for caregivers and productivity losses of paid and unpaid jobs are also understandably higher. However, this surplus of urgent care seems to last relatively short due to seemingly stabilized costs 4 and 12 months after the first FLS visit. Additional overlap in productivity loss of unpaid work and receiving homecare could have occurred.

However, the results should be interpreted with some caution. Firstly, due to lack of information for surgery costs, the DBC for surgical healthcare consumption did not include the operation itself and the costs of fractures (such as the hip) are therefore underestimated. Secondly, as the DBCs for surgical healthcare consumption and FLS care were calculated simultaneously to the self-reported data, patients could perhaps have reported additional visitations with medical specialists, paramedics, etc., while in reality they received diagnostic workup that was already included in the DBC costs. On the other hand, underestimation of self-reported visitations to healthcare professionals due to the recall period of 4 months could have occurred. Secondly, the societal costs, and therefore healthcare costs, do not include medication costs or additional technical examinations such as laboratory tests or X-rays related to fracture healing. As most patients, except for two patients who did not initiate treatment, initiated alendronate as AOM and the (generic) cost of alendronate is estimated at €37.46 per 3 months in the Netherlands [20], limited influence on the healthcare and societal costs could be expected. Lastly, instead of detailed questions inquiring about the amount of specialty care consumed across various types, a general question was opted for to determine the total number of specialty care and thereby limiting patient burden. A distinction on type of specialty care could therefore not be made.

Interestingly, our study did not report a significant difference according to fracture type. Notwithstanding 4 and 12 months after the first FLS visit these patients had similar costs compared to patients with other fractures, except for this having experienced a hip or clinical vertebral fracture.

Although this study fills the knowledge gap by assessing the societal pre- and post-FLS using data from a clinical study, there were some potential limitations. Firstly, the sample of this study did not present all patients with a fracture and osteoporosis as individuals who attend FLS care as our patient population did not include patients with osteopenia or patients who do not need AOM treatment. Moreover, our sample may not represent all patients with osteoporosis attending FLS, as this study was conducted solely in two centers in the Netherlands and only patients that agreed to participate in a clinical trial study were included. Difference in patient characteristics and healthcare access may vary in different countries.

Additionally, it is important to note the limited interpretability of the results. Although the study showed a significant decrease in societal costs before and after the first FLS visit, this reduction may not be solely attributed to the FLS and could be a natural cause of disease development. Future research investigating the impact of FLS on overall fracture costs will have to include a control arm such as the absence of FLS or another FLS setup.

Nonetheless, the data presented in this study provide costs based on real-world data in a longitudinal manner. This representation could be specifically interesting to assess more accurately the cost-effectiveness of FLS as most studies to date used simulation models. Moreover, as most studies present a lifetime or 2- to 10-year follow-up periods, the immediate economic representation in this study offers insights into the short-term implications of FLS. Therefore, more targeted assessments of the initial economic impact could facilitate implementation of FLS in clinics, research allocation associated with FLS, and scaling of FLS initiative efficiently, ultimately ensuring widespread accessibility based on patients’ needs. Future research could focus on difference in costs in patients undergoing surgery compared to conservative treatment without surgery and the impact of innovative AOM and treatment strategies.

## Conclusion

Societal costs before and 1 year after the first FLS visit significantly decreased over time. This study provides insights into the breakdown of FLS costs and primary drivers of FLS that could help implementation of FLS, as well as future cost-effectiveness studies investigating the potential benefits of FLS.

## Supplementary Information

Below is the link to the electronic supplementary material.

Supplementary file1 (DOCX 66 KB)

## Data Availability

Additional data that supports the study's findings can be made available on request from the corresponding author.
