# Supplementary material for: Societal costs before and up to 1 year after the first fracture liaison service visit in patients requiring anti-osteoporosis treatments
Source: Arch Osteoporos. 2024 May 13;19(1):36. doi: 10.1007/s11657-024-01390-7 (PMC11090956; doi:10.1007/s11657-024-01390-7)
Supplement: Supplementary file 1 — Supplementary file1 (DOCX 66 KB) [file 11657_2024_1390_MOESM1_ESM.docx]

*Supplementary Table 1: patient characteristics per FLS center*

|  | FLS MUMC+ (n=94) | FLS Viecuri (n=32) |
| --- | --- | --- |
| Age (mean ± SD) | 71 ± 12 | 73 ± 10 |
| Female (freq) | 78 (83%) | 22 (69%) |
| Education – college level (higer vocational/university) | 26 (28%) | 8 (25%) |
| Major fracture (freq) | 56 (59.6%) | 16 (50%) |
| Surgery (freq) | 25 (27%) | 7 (22%) |
| Time from fracture to first FLS (mean months ± SD) | 4.1 ± 2.6 | 2.5 ± 1.8 |
| % with surgery >4 months before first FLS | 11 (44%) | 1 (14%) |
| Recurrent fracture during trial (freq) | 9 (10%) | 8 (25%) |

*Supplementary Table 2: Missing values matrix of all variables (x-axis) and individual observations or rows in the dataset (y-axis). The cells in the plot indicate missing (pink) or complete (blue) data. Top horizontal axis represent all variables, bottom horizontal axis represent total missing cases per variable, left vertical axis represent total observations with the complete/missing pattern of the respective row, right vertical axis represents total missing values of the respective row.*


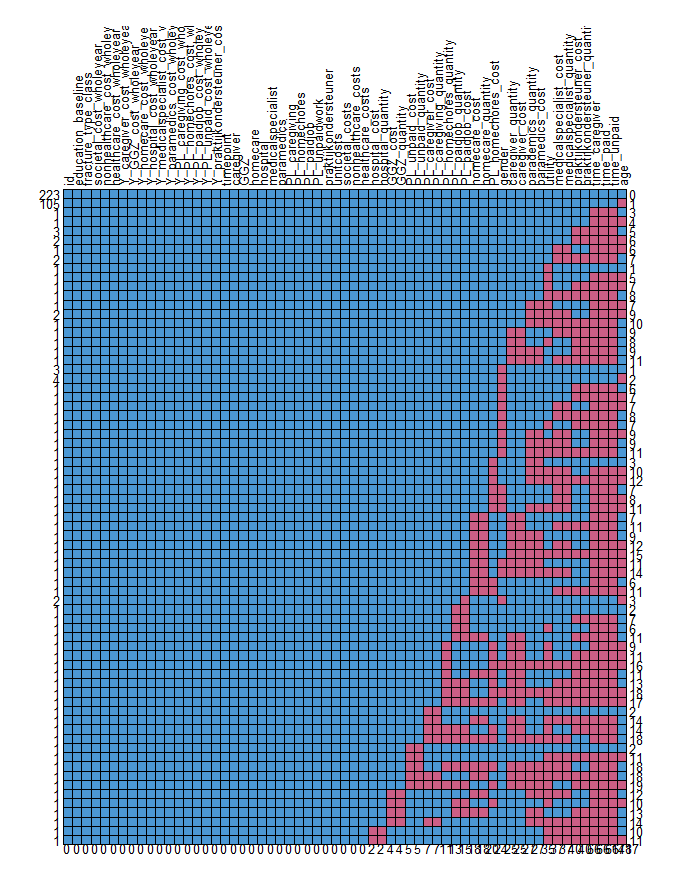


Supplementary Table 3: Effect of societal and healthcare costs over time including linear mixed regressions adjusted for confounding factors. The beta coefficient of the linear mixed regressions demonstrate the mean difference between the first measurement of four months before the first FLS visit and the time point tested (4 or 12 months after the first FLS visit). Sensitivity analysis adjusted for all possible confounding factors analyzing the effect of societal and healthcare costs over time including linear mixed regressions; CI, confidence interval; SD, standard deviation; coef, coefficient

| Linear mixed models | | Mean unit costs ± SD | Mean difference (95% CI) | p-value |
| --- | --- | --- | --- | --- |
| Societal costs over time | 4 months | €178 ± €341 | -604 (-985 to -224) | **0.002** |
|  | 12 months | €145 ± €405 | -696 (-1117 to -276) | **0.001** |
| Healthcare costs over time | 4 months | €121 ± €248 | -371 (-524 to -218) | **0.000** |
|  | 12 months | €109 ± €195 | -388 (-570 to -207) | **0.000** |

Supplementary Table 4: Effect of fractures and time on societal and healthcare costs including linear mixed regressions adjusted for confounding factors. The beta coefficient of the linear mixed regressions demonstrate the mean difference between the first measurement of four months before the first FLS visit and the time point tested (4 or 12 months after the first FLS visit). The beta coefficient of solely cost and fracture type (without specified time point) provides the aggregate effect of fracture type as variable; CI, confidence interval; SD, standard deviation; coef, coefficient

| Linear mixed models | | Mean unit costs ± SD | | Mean difference  ( 95% CI) | p-value |
| --- | --- | --- | --- | --- | --- |
|  |  | Major | Other |  |  |
| Societal costs and fracture type | | €362 ± €933 | €344 ± €519 | 219 (-162 to 599) | 0.257 |
|  | 4 months | €161 ± €196 | €206 ± €413 | -174 (-553 to 204) | 0.366 |
|  | 12 months | €140 ± €246 | €157 ± €239 | -217 (-636 to 201) | 0.307 |
| Healthcare costs and fracture type | | €257 ± €832 | €251 ± €414 | 78 (-88 to 245) | 0.354 |
|  | 4 months | €109 ± €139 | €142 ± €348 | -47 (-208 to 115) | 0.569 |
|  | 12 months | €107 ± €216 | €114 ± €167 | -81 (-272 to 111) | 0.406 |

Supplementary Table 5: Sensitivity analysis adjusted for all possible confounding factors analyzing the effect of societal and healthcare costs over time including linear mixed regressions. The beta coefficient of the linear mixed regressions demonstrate the mean difference between the first measurement of four months before the first FLS visit and the time point tested (4 or 12 months after the first FLS visit). Sensitivity analysis adjusted for all possible confounding factors analyzing the effect of societal and healthcare costs over time including linear mixed regressions; CI, confidence interval; SD, standard deviation; coef, coefficient

| Linear mixed models | | Mean unit costs ± SD | Mean difference (95% CI) | Sensitivity analysis  P value |
| --- | --- | --- | --- | --- |
| Societal costs over time | 4 months | €178 ± €341 | -604 (-992 to -226) | **0.000** |
|  | 12 months | €145 ± €405 | -696 (-1131 to -153) | **0.001** |
| Healthcare costs over time | 4 months | €121 ± €248 | -370 (-525 to -211) | **0.000** |
|  | 12 months | €109 ± €195 | -401 (-580 to -188) | **0.001** |

Supplementary Table 6: Sensitivity analysis adjusted for all possible confounding factors analyzing the effect of fractures and time on societal and healthcare costs including linear mixed regressions adjusted for confounding factors. The beta coefficient of the linear mixed regressions demonstrate the mean difference between the first measurement of four months before the first FLS visit and the time point tested (4 or 12 months after the first FLS visit). The beta coefficient of solely cost and fracture type (without specified time point) provides the aggregate effect of fracture type as variable; CI, confidence interval; SD, standard deviation; coef, coefficient

| Linear mixed models | | Mean unit costs ± SD | | Mean difference  ( 95% CI) | Sensitivity analysis  P value |
| --- | --- | --- | --- | --- | --- |
|  |  | Major | Other |  |  |
| Societal costs and fracture type | | €362 ± €933 | €344 ± €519 | 216 (-168 to 599) | 0.232 |
|  | 4 months | €161 ± €196 | €206 ± €413 | -172 (-553 to 207) | 0.372 |
|  | 12 months | €140 ± €246 | €157 ± €239 | -211 (-632 to 213) | 0.259 |
| Healthcare costs and fracture type | | €257 ± €832 | €251 ± €414 | 75 (-94 to 245) | 0.265 |
|  | 4 months | €109 ± €139 | €142 ± €348 | -47 (-209 to 115) | 0.514 |
|  | 12 months | €107 ± €216 | €114 ± €167 | -77 (-269 to 117) | 0.331 |
